# Impact of a Mindfulness-Based Stress Reduction Program on Psychological Well-Being, Cortisol, and Inflammation in Women Veterans

**DOI:** 10.1007/s11606-022-07584-4

**Published:** 2022-08-30

**Authors:** Karen L. Saban, Eileen G. Collins, Herbert L. Mathews, Fred B. Bryant, Dina Tell, Beverly Gonzalez, Sudha Bhoopalam, Christopher P. Chroniak, Linda Witek Janusek

**Affiliations:** 1 Center of Innovation for Complex Chronic Healthcare, Edward Hines Jr. VA, 5000 S. 5th Ave., Hines, IL 60141 USA; 2 Marcella Niehoff School of Nursing, Loyola University Chicago, 2160 S. First Ave, Center for Translational Research and Education, Maywood, IL 60153 USA; 3 Department of Biobehavioral Nursing Science, College of Nursing, University of Illinois Chicago, 845 S. Damen Ave. MC 902, Chicago, IL 60612 USA; 4 Stritch School of Medicine, Loyola University Chicago, 2160 S. First Ave, Center for Translational Research and Education, Maywood, IL 60153 USA; 5 Department of Psychology, Loyola University Chicago, 1032 W. Sheridan Road, Coffey Hall Rm. 242, Chicago, IL 60660 USA; 6 The Insight Center, 122 S. Michigan Ave. Suite 1457, Chicago, IL 60603 USA

**Keywords:** veteran, women, mindfulness-based stress reduction, randomized controlled trial, diurnal salivary cortisol, cytokines

## Abstract

**Background:**

Women veterans experience higher levels of stress-related symptoms than their civilian counterparts. Psychological stress is associated with greater inflammation and may increase risk for cardiovascular disease (CVD). Mindfulness-based stress reduction (MBSR) has been found to improve psychological well-being in other populations but no randomized controlled trials (RCT) have been conducted examining the impact of MBSR on well-being and inflammation in women veterans at risk for CVD.

**Objective:**

Determine the effectiveness of MBSR in improving psychological well-being, cortisol, and inflammation associated with CVD in women veterans.

**Design:**

The design is a RCT comparing MBSR to an active control condition (ACC) consisting of a health education program.

**Participants:**

Women veterans (*N*=164) with risk factors for CVD from the Chicagoland area participated in the study.

**Intervention:**

An 8-week MBSR program with weekly 2.5-h classes was compared to an ACC consisting of an 8-week health promotion education program with weekly 2.5-h classes.

**Main Measures:**

The outcomes were psychological well-being [perceived stress, depressive symptoms, loneliness, and post-traumatic stress disorder (PTSD)] symptoms and stress-related markers, including diurnal salivary cortisol and cytokines interleukin-6 (IL-6) and interferon gamma (IFN-γ). Data were collected at baseline, 4 weeks (mid-point of intervention), 8 weeks (completion of intervention), and 6 months after completion of MBSR or ACC.

**Key Results:**

Compared to the ACC, women who participated in MBSR reported less perceived stress, loneliness, and symptoms of PTSD. Although there were no significant differences between groups or changes over time in IL-6 or IFN-γ, participants in the MBSR program demonstrated a more rapid decline in diurnal salivary cortisol as compared to those in the ACC.

**Conclusions:**

MBSR was found to improve psychological well-being and decrease diurnal salivary cortisol in women veterans at risk for CVD. Health care providers may consider MBSR for women veterans as a means by which to improve their psychological well-being.

Women veterans experience higher levels of stress-related symptoms as well as higher prevalence of mental health disorders, such as depression and post-traumatic stress disorder (PTSD) as compared to their civilian women counterparts.^1–3^ A multitude of factors may place women veterans at higher risk for stress and mental health disorders including greater exposure to lifetime traumatic stressors, such as early life adversity, sexual trauma, physical assault, and combat exposure.^4,5^ Compelling evidence implicates psychological stress in the etiology and pathogenesis of atherosclerosis and it is increasingly evident that chronic stress may promote inflammatory-based diseases, such as cardiovascular disease (CVD) and stroke.^6–8^ Yet few psychobehavioral interventions have been studied for their ability to reduce psychological distress and inflammation associated with CVD risk in women veterans.

Meditation-based interventions, such as mindfulness-based stress reduction (MBSR), may benefit cardiovascular health, including prevention of CVD.^9,10^ MBSR, developed by Kabat-Zinn,^11^ involves intensive training in mindfulness, which promotes positive adaptation to life stress. Practitioners of MBSR gain increased awareness and insight into the relationship between their thoughts, emotions, and somatic reactivity, which can facilitate change in conditioned patterns of emotional reaction. These skills can be applied to prevent disease or manage the stress of living with disease or other types of stressors.

Meta-analyses of MBSR and health benefits reveal consistent and strong effect sizes for psychological benefits in individuals dealing with emotional distress.^12–14^ Studies in non-veteran populations demonstrate that MBSR decreases anxiety, depression, and loneliness while improving biologic markers of stress, such as cortisol and inflammatory cytokines.^15–17^ For example, findings from a randomized controlled trial (RCT) of women newly diagnosed with breast cancer reveal MBSR to decrease perceived stress and depressive symptoms along with lowering markers of inflammation associated with stress, including circulating levels of cytokines, interleukin-6 (IL-6), and tumor necrosing factor-alpha (TNF-α), while enhancing production of interferon gamma (IFN-γ).^16^

Findings from the few studies of MBSR in veteran populations revealed MBSR to reduce symptoms of depression and lower diurnal salivary cortisol, a stress-related hormone, in veterans experiencing post-traumatic stress disorder (PTSD).^18,19^ Similarly, others reported MBSR to improve PTSD symptoms in veterans.^20,21^ However, no studies were found that examined MBSR in women veterans to determine the efficacy in reducing symptoms and biological indicators of stress (e.g., diurnal salivary cortisol and inflammatory cytokines). In addition, women have been underrepresented in studies examining MBSR in veterans with a recent meta-analysis finding that 85% of the sample across all studies reviewed were male.^22^ Furthermore, findings from a qualitative study suggest that women veterans prefer “women-only” MBSR groups.^23^ Therefore, the aim of this RCT was to examine the effectiveness of MBSR to reduce perceived stress, depressive symptoms, loneliness, and symptoms of PTSD, and to lower diurnal salivary cortisol and inflammatory cytokines in women veterans at risk for CVD.

## METHODS

### Study Design

The design was a RCT with an active control condition (ACC) group. The trial was registered at ClinicalTrials.gov, identifier NCT01784796. The study was approved by the sponsoring Institutional Review Board (IRB) and all participants provided informed consent prior to beginning the study. No adverse events were reported.

### Participants

Participants were eligible if they self-identified as a woman veteran receiving care at the VA Medical Center, were 18 years of age or older, and had at least one of the following risk factors for CVD: (1) body mass index (BMI) ≥ 25, (2) total cholesterol ≥240, (3) diagnosed with diabetes mellitus or pre-diabetic, (4) systolic blood pressure >120 and/or diagnosed with hypertension and/or taking antihypertensive medication, and (5) reported parental history of myocardial infarction (myocardial infarction) prior to age 60. Participants were excluded if they reported a history of MI, ischemic heart disease/coronary artery disease, left ventricular hypertrophy, or ischemic stroke. In addition, women were excluded if they were pregnant, planned on becoming pregnant during the study, gave birth in prior 6 weeks, or were lactating during the study period, had prior MBSR training, active infection, major autoimmune disorders requiring the use of immune suppressant medication, current cancer, history of suicide attempt (s) in past year, or were unable to participate in a group setting without feeling uncomfortable. In addition to posting flyers, informational letters were sent to potential participants from two Midwest VA medical centers who met general study criteria based on chart review. Data collection took place between July 2013 and June 2018. Estimated sample size was based on a small effect size of MBSR on stress and depressive symptoms in previous studies.^24–27^

### Randomization

**Figure 1:**
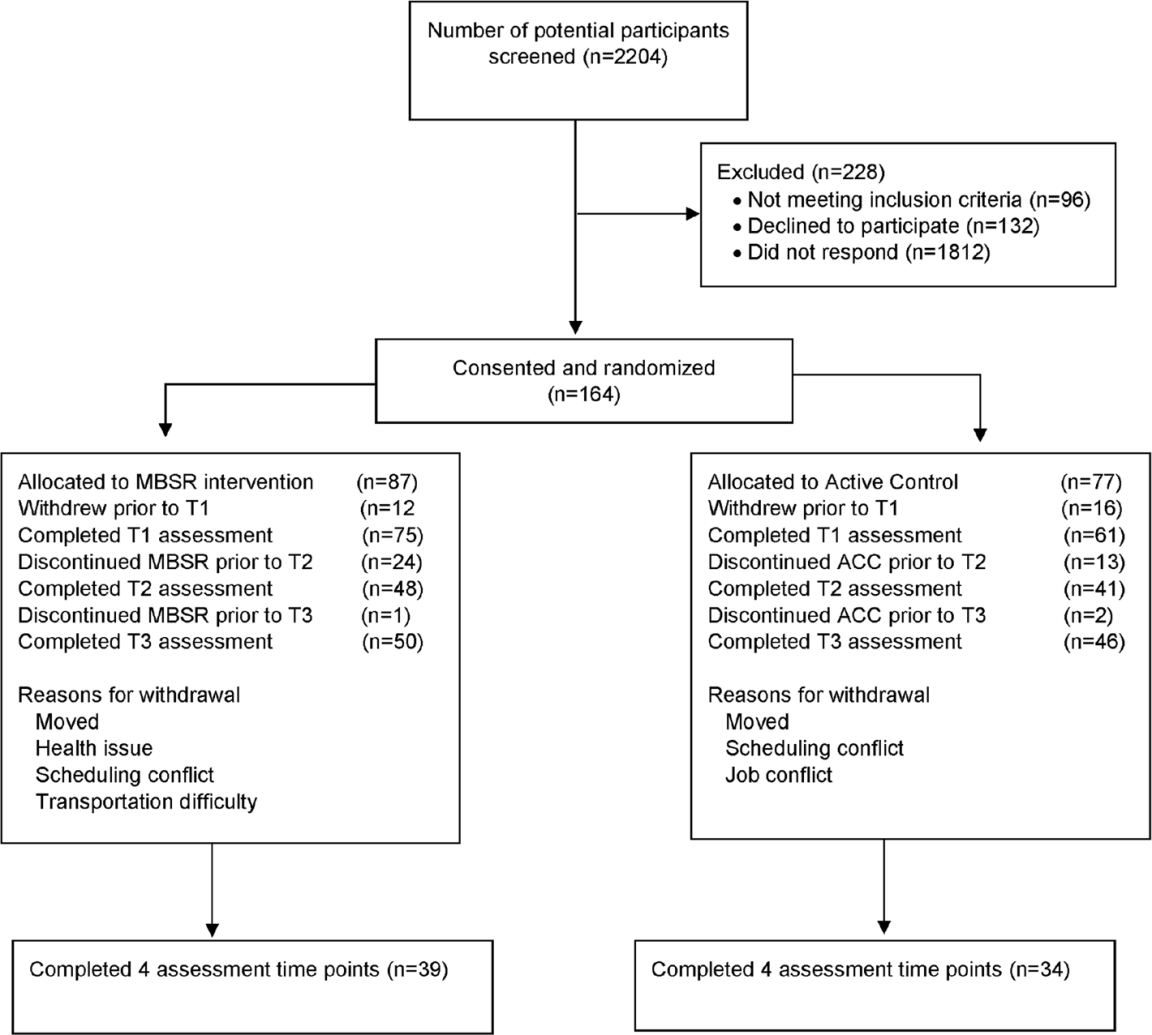
Flow diagram of the recruitment process and the progress through the phases of the study. MBSR, mindfulness-based stress reduction; ACC, active control condition. T1 – baseline, T2 – intervention mid-point, T3 – intervention completion, T4 – 1 month post-completion of the intervention. Note that number of participants who completed T3 is higher than those who completed T2 because of missing completed surveys at T2.

Participants (*n*=164) were randomized to either MBSR or ACC (described below) using stratified block randomization with a random number generator based on age categories (categorized as years 18–24, 25–34, 35–45, 46–59, and 60 years of age and older). See CONSORT (Figure 1).

### MBSR Intervention and Active Control Condition

#### MBSR Intervention

MBSR, based on that originally developed by Kabat-Zinn,^11^ consisted of an 8-week, group-based standardized program focused on mindfulness meditation, body awareness, and gentle yoga. Participants attended weekly 2.5-h classes with 4 to 8 women per group. All classes were led by the same licensed clinical psychologist who was an experienced MBSR instructor.

#### Active Control Condition (ACC)

The ACC, which ran concurrently with the MBSR program, consisted of an 8-week, group-based health promotion education program with topics including (1) cooking with organic foods, (2) body mechanics to protect the back, (3) communicating with health providers, (4) enhancing memory, (5) using MyHealtheVet (patient medical record) and health screening, (6) growing an herb garden, (7) over-the-counter medication safety, and (8) exploring healthy hobbies. Similar to the MBSR program, participants met weekly for 2.5-h in small groups (4 to 8 participants). Topic experts taught the classes (i.e., nutritionist, physical therapist) using standardized content.

### Procedures

Participants completed a self-administered written questionnaire and had 5cc of blood drawn for measurement of IL-6 and IFN-γ at baseline (T1), 4 weeks (mid-way through intervention) (T2), 8 weeks (at the completion of the intervention) (T3), and 6 months after completion of the intervention (T4). For each time point, participants collected saliva samples using a Salivette (Sarstedt, Nϋmbrecht, Germany) at awakening, 30 min after awakening, at lunchtime, dinner time, and bedtime for two consecutive days. Participants kept samples refrigerated in their homes and brought samples to the clinic the day following collection.^28^ Participants were provided stipends for each data collection ($50 each) and each class attended ($15 each).

### Outcomes

#### Perceived Stress Scale (PSS)

The PSS is a measure of the degree to which situations in one’s life are considered to be stressful.^29^ It contains ten questions with five responses on a Likert-type scale. Scores range from 0 to 40 with higher scores indicating higher levels of perceived stress. In the present sample, Cronbach’s alphas ranged from .89 to .92.

#### Center for Epidemiologic Studies, Depression Scale (CES-D)

The CES-D is a 20-item scale that measures the respondents’ level of depressive symptoms with a 4-point Likert-type scale.^30^ Scores range from 0 to 60 with higher scores indicating greater depressive symptoms. In the present sample, Cronbach’s alphas ranged from .92 to .95.

#### UCLA Loneliness Scale

The UCLA Loneliness Scale is a 20-item scale that assesses the subjective level of social isolation using a 4-point Likert-type scale.^31^ Scores range from 0 to 60 with higher scores suggesting greater loneliness. In the present sample, Cronbach’s alphas ranged from .81 to .95.

#### PTSD Checklist Civilian Version (PCL-C)

The PCL-C is a 17-item survey that measures the severity of DSM-4 PTSD symptoms using a 5-point Likert scale.^32^ Total scores range from 0 to 80 with higher scores indicating greater PTSD symptom severity. In the present sample, Cronbach’s alphas ranged from .95 to .97.

#### Diurnal Salivary Cortisol

A commercial enzyme immunoassay (EIA) kit (Salimetrics, LLC, State College, PA) was used to measure diurnal salivary cortisol. Samples were assayed in duplicate. The sensitivity of the assay was <0.0007 μg/dl and the coefficient of interassay variability ranged from 0.012 to 3.000 μg/dl. The area under the curve (AUC) was calculated to reflect the total daily cortisol level.

#### Cytokines

##### Isolation of Peripheral Blood Mononuclear Cells (PBMCs)

Heparinized blood was processed immediately in a laboratory overlaid into Ficoll/Hypaque and centrifuged at 1000 × *g* for 20 min. PBMCs at the interface were washed twice with Hank’s balanced salt solution prior to measurement of cytokine production as previously described.^33^

##### PBMC Cytokine Production

PMBCs were isolated, as described previously.^33^ PBMCs (1×10^6^ cells/ml) were cultured with and without PMA/PHA (PMA at 20 ng/well; PHA at 0.05%/well) in 24-well plates for 48 h at 37 °C. Aliquots of the culture supernatants were stored at −80° C for subsequent analysis of IL-6 and IFN-γ production.

##### Cytokine Measurement (ELISA)

All cytokines were measured using quantitative sandwich enzyme assay kits (Quantikine kits, R&D Systems, Minneapolis, MN). Sensitivities for cytokines were IL-6 <0.7 pg/ml and IFN-γ <3 pg/ml. The coefficient of variation ranged from 2.6 to 4.9%.

### Covariates

#### Social Provisions Scale (SPS)

The SPS is a 24-item instrument that measures attachment, social integration, reassurance of worth, reliable alliance, guidance, and opportunity for nurturance.^34^ The total score ranges from 24 to 96 with higher scores indicating greater social support. In the present sample, Cronbach’s alphas ranged from .78 to .93.

#### Combat Exposure Scale (CES)

The CES is a 7-item tool that assesses wartime stressors experienced by veterans. Items are rated on a 5-point frequency scale.^35^ Scores range from 0 to 41 with higher scores suggesting greater combat stress. In the present study, internal consistency was good (Cronbach’s alpha’s .78–.89).

#### Demographic and Medical History

Age, race, education level, employment status, household income, and marital status were self-reported on a written questionnaire. In addition, smoking status and comorbidities were self-reported. Body mass index (BMI) was calculated based on weight and height measured using standard equipment.

### Assessment of Effectiveness of Intervention

#### Five-Facet Mindfulness Questionnaire (FFMQ)

The FFMQ is a 39-item tool that assesses five facets of mindfulness: observing, describing, acting with awareness, non-judging of inner experience, and non-reactivity to inner experience.^36^ Subscale scores range from 7 to 40 with higher scores indicating a greater presence of facet. In the present sample, internal consistency was high across subscales, with Cronbach’s alphas .85–.97.

### Statistical Analysis

Preliminary analyses were carried out using IBM SPSS 27.0 (Chicago, IL). To test for differences between the groups for demographic variables, *t*-tests and *χ*^2^ tests were conducted as appropriate.

HLM 8.01 software for computing multilevel model for change,^37^ based on full maximum likelihood estimation, was used to examine intra-individual (within-subject) and inter-individual (between-subject) differences at baseline and trajectories of change over time in psychobehavioral outcomes (perceived stress, depressive symptoms, loneliness, and PTSD symptoms), inflammatory (IL-6 and IFN-γ production), and salivary cortisol computed as AUC. Data were analyzed using intent-to-treat approach, as growth curve modeling techniques allow for the analysis of participants with incomplete data across time points.^38^

With HLM of longitudinal data, the outcome variables are conceptualized to be nested within individuals, and time is treated as a continuous variable.^37^ In the present analysis, time was measured in weeks from T1. T1 was coded as zero; hence, the slope coefficients are interpreted as a change per each additional week from T1. Both linear and quadratic patterns of change were examined; given the goodness-of-fit tests of the deviance, a linear trend was the most appropriate fit for psychological and inflammatory outcomes and a model with a quadratic slope was a better fit for change in cortisol.

The HLM analysis for each of the outcome variables was performed in two stages. The first stage of HLM analysis examined the potential effects of the demographic variables (age, race, education, income, marital status), health behaviors (BMI, smoking), comorbidities, and number of sessions attended. Variables that were associated with either the intercept or the slope parameters (using *p* ≤.10, to be more conservative) were included in the second stage of analysis and retained in the final model for each outcome. In addition, all models controlled for combat exposure and social support, and models for cortisol and immune outcomes controlled for BMI.

During the second stage, the grouping variable (MBSR/ACC) and covariates identified in the first stage of analysis were entered into the model simultaneously. To help interpret the fixed effects, continuous predictor variables were grand mean centered (i.e., the variable’s sample mean was subtracted from each observation). The standardized effect sizes for polynomial trend were computed using the formula, *δ* = *β*_11_/ √ *τ*_11_.^39^ To examine whether group differences were statistically significant at T4, post hoc analyses were performed for each outcome, by re-centering the variable of time to the last assessment.^40^

## RESULTS

### Descriptive Characteristics of Participants

Table 1 reports summary descriptive statistics of the demographic characteristics of the women. No differences were found between women in the MBSR intervention compared to women in the ACC. Psychological measure results across assessment times are provided in Table 2.

**Table 1:** Demographic Characteristics of Women in MBSR and ACC Group

	MBSR (*n*=75)	Active control (*n*=61)	*p-*values
Age (*yrs*) mean ± SD	50.83 ± 10.79	50.51 ± 10.42	.855
Education	Percent (%)		.476
High school or GED	2.8	–	
Some college	37.5	45.6	
College graduate	34.7	38.6	
Post-college degree	25.0	15.8	
Race			.550
Caucasian	55.8	49.2	
African American	39.0	42.9	
Asian	–	1.6	
Hawaiian or Pacific Islander	1.3	1.6	
Other	3.9	4.8	
Ethnicity			.595
Not Hispanic	85.9	89.1	
Latinix	14.1	10.9	
Marital status			.780
Married	33.3	28.1	
Divorced/separated/widowed	40.3	45.6	
Single/never married	26.4	26.3	
Income			.952
Less than $25,000	46.5	41.8	
$25,001–50,000	28.2	23.6	
50,001–$75,000	15.5	20.0	
>$75,000	10.0	14.6	
BMI	27.34 (14.25)	25.33 (23.93)	.513
Combat exposure	4.39 (6.59)	3.69 (7.45)	.583

Values are based on the sample at T1

To test for differences between MBSR and ACC groups, *t*-tests and *χ*2 tests were conducted as appropriate

*SD* standard deviation

**Table 2:** Descriptive Statistics for Psychosocial and Biological Variables

Variable	Time 1		Time 2		Time 3		Time 4	
	*Mean*	*SD*	*Mean*	*SD*	*Mean*	*SD*	*Mean*	*SD*
Active control group								
PSS	17.50	7.52	16.40	7.238	15.50	7.33	14.35	6.23
UCLA-LS	46.58	10.86	44.46	12.683	43.44	10.94	45.00	9.70
PCL-C	42.26	17.85	33.04	14.432	32.92	14.89	36.46	16.86
CES-D	19.22	11.67	15.57	12.414	14.46	11.03	15.29	12.52
SPS	73.35	11.45	73.67	10.467	75.86	10.77	73.14	10.37
CES	3.69	7.44	–	–	–	–	–	–
IL-6 production	8.99	1.14	9.02	0.81	8.84	0.74	8.93	1.01
INF-γ production	8.31	1.15	8.29	1.32	8.26	1.08	8.38	1.19
Cortisol (AUC)	0.14	0.29	0.11	0.12	0.11	0.09	0.16	0.16
MBSR group								
PSS	19.64	8.21	17.45	8.54	15.94	8.53	16.63	8.52
UCLA-LS	48.03	14.83	48.32	13.85	45.22	13.20	44.31	15.24
PCL-C	48.56	18.79	42.33	20.27	42.49	18.70	37.10	18.20
CES-D	22.78	13.76	20.61	13.34	18.38	13.26	18.66	14.49
SPS	73.54	13.13	71.52	14.37	74.56	18.00	73.57	14.79
CES	4.39	6.57	–	–	–	–	–	–
IL-6 production	9.17	1.01	9.39	0.82	8.89	1.00	9.17	0.84
INF-γ production	8.57	1.25	8.57	1.25	8.49	1.03	8.61	1.36
Cortisol (AUC)	0.10	0.04	0.09	0.07	0.07	0.04	0.07	0.04

Abbreviations: *PSS* Perceived Stress Scale; *UCLA-LS* Loneliness Scale; *PCL-C* PTSD Checklist Civilian Version; *CES-D* The Center for Epidemiologic Studies Depression; *SPS* Social Provisions Scale; IL-6 and INF-γ are pg/ml of plasma production; *AUC* area under the curve; *SE* standard error of the coefficient

In the MBSR intervention group, 54% of women attended six or more sessions, 25% of women attended one to five sessions, and 21% of women did not attend any sessions. In the ACC, 49% of women attended six or more sessions, 27% of women attended one to five sessions, and 24% of women did not attend any sessions. No group differences were found between women in MBSR (M=4.48, SD=0.36) and ACC (M=4.20, SD=0.36) in the total number of classes they attended (*t*(142)=0.48, *p*=.63).

### Effects of MBSR Intervention

#### Mindfulness Outcomes

**Figure 2:**
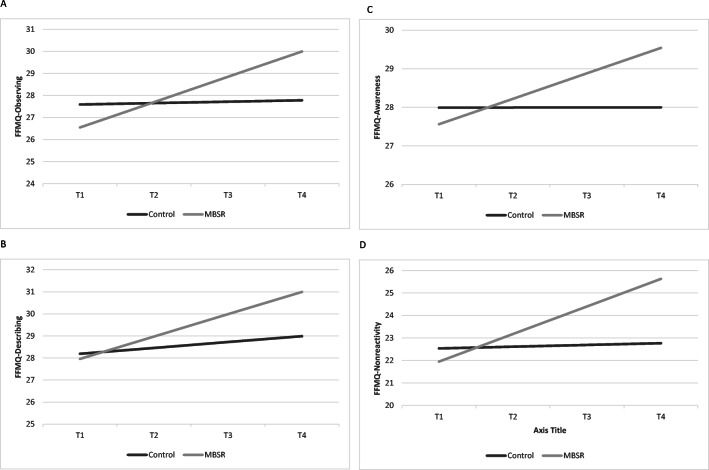
**a–d** Graphical representation of the effects of mindfulness-based stress reduction (MBSR) intervention and control condition on mindfulness outcomes as assessed by the Five-Facet Mindfulness Scale (FFMS). Graphs are estimated by the hierarchical linear models from the baseline (before the intervention; T1) to 6 months post-completion of the intervention (i.e., T4). The solid grey line represents the group of women who were randomized into the control group; the solid black line represents the group of women who were randomized into MBSR intervention; **a** Participation in MBSR intervention was associated with a steeper linear change in observing, indicating a greater increase of this mindfulness facet as compared to women who were randomized into the control group (*b* = 0.2425, SE = 0.0953, *p* =.01); **b** Participation in MBSR intervention was associated with a steeper linear change in describing, indicating a greater increase of this mindfulness facet as compared to women who were randomized into the control group (*b* = 0.1643, SE = 0.0875, *p* =.043); **c** Participation in MBSR intervention was associated with a steeper linear change in awareness, indicating a greater increase of this mindfulness facet as compared to women who were randomized into the ACC group (*b* = 0.2031, SE = 0.0951, *p* = .02); **d** Participation in MBSR intervention was associated with greater increase in non-reactivity to inner experience for women facet of mindfulness in MBSR as compared to ACC group (*b* = 0.1971, SE = 0.0935, *p* =.03, *δ* = 0.85).

Women who received MBSR training had a significant increase in observing (*b* = 0.2425, SE = 0.0953, *p* =.01, *δ* =2.01), describing (*b* = 0.1643, SE = 0.0875, *p* =.043, *δ* =1.61), awareness (*b* = 0.2031, SE = 0.0951, *p* = .02, *δ* =2.48), and non-reactivity to inner experience (*b* = 0.1971, SE = 0.0935, *p* =.03, *δ* = 0.85) (Table 3). Post hoc analysis of group differences at T4 revealed significant differences in awareness and non-reactivity to inner experience (*p*-values < .04) between women in MBSR and ACC (Figure 2a–d).

**Table 3:** Final Hierarchical Linear Model Estimation of the Fixed Effects for Five Facets of Mindfulness Questionnaire

	FFMQ-Observing		FFMQ-Describing		FFMQ-Awareness		FFMQ-Non-reactivity		FFMQ-Non-judge	
	*β*	SE	*β*	SE	*β*	SE	*β*	SE	*β*	SE
Fixed effects: baseline										
Intercept	27.7134	0.8567	29.0238	0.9670	29.2375	0.9364	22.9521	0.7700	30.4565	1.0031
Group	−1.6396	1.0708	−2.3257	1.1534	−3.8513	1.1179	−2.8063	0.9755	−1.1196	1.2024
Age	−0.0294	0.0462	0.0672	0.0547	0.1134	0.0532	0.0346	0.0419	0.0054	0.0572
Combat exposure	0.2508	0.0691	0.0983	0.0840	−0.0594	0.0814	−0.0025	0.0634	−0.1188	0.0882
Social support	0.1174	0.2336	0.7992	0.2347	0.7276	0.2276	0.3682	0.2165	1.1493	0.2457
Time slope (linear)^†^										
Intercept	0.0794	0.0799	0.0940	0.0752	0.0117	0.0819	0.0367	0.0767	0.1829	0.0917
Group	0.2425^b^	0.0953	0.1643^c^	0.0875	0.2031^c^	0.0951	0.1971^c^	0.0935	−0.0807	0.1063
Age	−0.0020	0.0048	0.0015	0.0044	−0.0002	0.0048	0.0002	0.0047	0.0051	0.0054
Combat exposure	−0.0303	0.0090	−0.0105	0.0061	0.0025	0.0067	−0.0042	0.0072	0.0060	0.0075
Social support	0.0169	0.0192	0.0142	0.0183	0.0035	0.0200	0.0098	0.0180	0.0071	0.0225

Abbreviations: *FFMQ* Five Facets of Mindfulness Questionnaire, *SE* standard error of the coefficient

Age, combat exposure, and social support variables were grand mean centered

^†^Time was coded 0 at the first assessment visit

^a^*p* < .001; ^b^*p*< .01; ^c^*p*≤ .05

#### Psychological Outcomes

**Figure 3:**
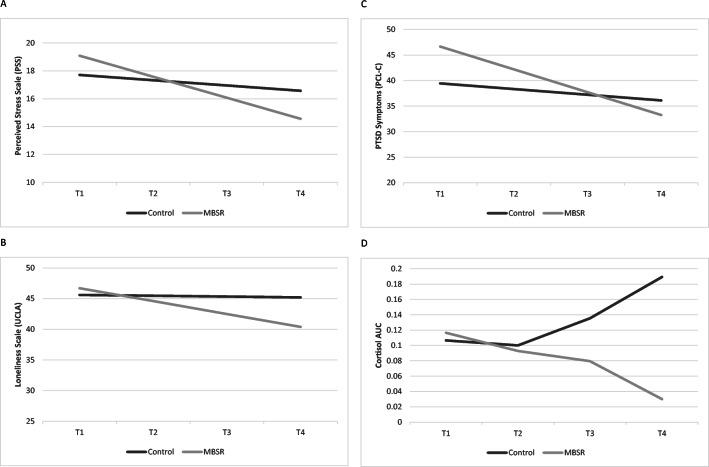
**a–d** Graphical representation of the effects of mindfulness-based stress reduction (MBSR) intervention and control condition on psychological outcomes and cortisol area under the curve (AUC). Graphs are estimated by the hierarchical linear models from the baseline (before the intervention; T1) to 6 months post-completion of the intervention (i.e., T4). The solid grey line represents the group of women who were randomized into the control group; the solid black line represents the group of women who were randomized into MBSR intervention; **a** Participation in MBSR was associated with a steeper linear change in perceived stress *b* = −0.2475, SE = .1241, *p* = .002), indicating faster improvement in these symptoms as compared to women who were randomized into the control group; **b** Participation in MBSR intervention was associated with a steeper linear change in loneliness (*b* = −0.2973, SE = 0.1451, *p* =.04), indicating faster improvement in these symptoms as compared to women who were randomized into the ACC group; **c** Participation in MBSR intervention was associated with a steeper linear change in PTSD symptoms (*b* = 0.8371, SE = 0.3245, *p* =.01), indicating faster improvement in these symptoms as compared to women who were randomized into the control group; **d** Participation in MBSR intervention was associated with a steeper quadratic change in cortisol AUC (*b*= −0.0017, SE= .00073, *p* = .02), indicating a more rapid decline in cortisol as compared to women who were randomized into the control group.

No significant group differences were found in perceived stress, depressive symptoms, loneliness, and PTSD symptoms between groups at baseline before the intervention. Participation in the MBSR intervention was associated with a steeper linear change in perceived stress (*b* = −0.2475, SE = .1241, *p* = .002, *δ* = .65), loneliness (*b* = −-0.2973, SE = 0.1451, *p* =.04, *δ* = .83), and PTSD symptoms (*b* = 0.8371, SE = 0.3245, *p* =.01, *δ* = 1.8), indicating faster improvement in these outcomes compared to women randomized to ACC (Figure 3a–c). No group differences were observed at baseline or linear slope in depressive symptoms (Table 4).

**Table 4:** Final Hierarchical Linear Model Estimation of the Fixed Effects for Psychological Outcomes

	PSS		UCLA-LS		PCL-C		CES-D	
	*β*	SE	*β*	SE	*β*	SE	*β*	SE
Fixed effects: baseline								
Intercept	15.7346	1.3043	41.2212	1.7742	45.7889	3.8437	28.5003	2.4062
Group	1.3704	1.3536	0.7675	1.8573	7.5478	3.2885	2.7584	1.9781
Age	−0.0958	0.0638	−0.0200	0.0877	−0.2836^c^	0.1425	−0.1471	0.0920
Race	1.4814	1.3563	−0.7471	1.8678	1.5405	3.1082	−0.6414	1.9908
Combat exposure	0.1562	0.1019	0.1218	0.1347	0.8231^a^	0.2097	0.4775^b^	0.1414
Social support	1.0071^a^	0.2783	3.4797	0.3889^a^	−0.4099^b^	0.1275	−0.5343^a^	0.0855
Time slope (linear)^†^								
Intercept	−0.1103	0.1218	−0.0600	0.1677	−0.4364	0.3939	−0.2921	0.2421
Group	−0.2475^c^	0.1241	−0.2973^c^	0.1451	−0.8371^b^	0.3245	−0.1531	0.2018
Age	0.0031	0.0061	0.0021	0.0088	0.0029	0.0162	−0.0054	0.0101
Race	−0.1799	0.1230	0.1475	0.1746	−0.0458	0.3209	−0.0225	0.1988
Combat exposure	0.0056	0.0098	0.0057	0.0125	0.0032	0.0211	0.0063	0.0142
Social support	0.0507	0.0270	−0.0484	0.0367	0.0118	0.0140	0.0073	0.0090

Abbreviations: *PSS* Perceived Stress Scale, *UCLA-LS* Loneliness Scale, *PCL-C* PTSD Checklist Civilian Version; *QLI* Quality of Life Index; *CES-D* The Center for Epidemiologic Studies Depression; *SE* standard error of the coefficient

Age, combat exposure, social support variables were grand mean centered

Race was a dichotomous variable (White=0/non-White=1)

^†^Time was coded 0 at the first assessment visit

^a^*p* < .001; ^b^*p*< .01; ^c^*p*≤ .05

#### Inflammatory Outcomes

Results revealed no differences in log-IL6 or log-INF-γ levels between women in the MBSR and ACC at baseline or in change over time. Greater BMI was associated with lower log IFN-γ at baseline (*b* = −0.0128, SE = 0.0060, *p* =.04, *δ* =0.02), but not log-IL-6 (Table 5).

**Table 5:** Final Hierarchical Linear Model Estimation of the Fixed Effects for Immune Outcomes and Cortisol (Area Under the Curve)

	IL-6 (log)		IFNg (log)		Cortisol (AUC)	
	*β*	SE	*β*	SE	*β*	SE
Fixed effects: *baseline*						
Intercept	8.5079	0.2178	7.9629	0.2682	0.1438	0.0459
Group	0.2893	0.1766	0.3221	0.2144	−0.0730	0.0482
Age	0.0023	0.0084	0.0043	0.0101	−0.0020	0.0024
BMI	−0.0055	0.0049	−0.0128^c^	0.0060	−0.0121	0.0480
Race	0.5734^b^	0.1789	0.5023^c^	0.2173	0.0062^a^	0.0016
Combat exposure	0.0105	0.0121	0.0084	0.0157	−0.0025	0.0036
Social support	0.0126	0.0076	0.0123	0.0093	0.00003	0.0022
Time slope (*linear*)†						
Intercept	0.024841	0.027768	−0.0103	0.0279	−0.0161	0.0105
Group	−0.023446	0.022679	0.0011	0.0224	0.0205^d^	0.0112
Age	−0.001060	0.001138	0.0018	0.0011	0.0008	0.0005
BMI	−0.000163	0.000597	−0.0003	0.0005	0.0011	0.0110
Race	−0.016589	0.023012	−0.0022	0.0229	−0.0016^a^	0.0003
Combat exposure	0.000002	0.001713	0.0022	0.0017	−0.00002	0.0008
Social support	−0.000682	0.001021	−0.00046	0.0010	−0.00011	0.0005
Time slope (*quadratic*)						
Intercept	–	–	–	–	0.0014	0.00068
Group	–	–	–	–	−0.0017^c^	0.00073
Age	–	–	–	–	−0.00006	0.00003
Race	–	–	–	–	−0.00016	0.00071
BMI	–	–	–	–	0.00011^a^	0.00002
Combat exposure	–	–	–	–	0.000001	0.00006
Social support	–	–	–	–	.0000001	0.0003

Abbreviations: IL-6 and INFg are pg/ml of plasma production. *SE* standard error of the coefficient

Age, combat exposure, social support variables were grand mean centered

Race was a dichotomous variable (White=0/non-White=1)

^†^Time was coded 0 at the first assessment visit

^a^*p* < .001; ^b^*p*< .01; ^c^*p*≤ .05

#### Cortisol

A non-linear model with a quadratic slope was a better fit to reflect the trajectory of change in cortisol (AUC) over the course of the study, compared to a linear model. No differences were found between the groups at baseline (T1) or in the linear slope (see Table 5). Women in the MBSR intervention group had a more rapid decline in cortisol (AUC) as indicated by the steeper quadratic slope (*b*= −0.0017, SE= .00073, *p* = .02, *δ* =0.71). A computation of the “peak” cortisol (AUC) revealed that for women in the MBSR group, cortisol started to decline more rapidly at 6 months, whereas for women in the ACC, cortisol started to rise (Figure 3d). Note that trajectories are based on the estimates computed by the regression models and are not group means.

## DISCUSSION

This is one of the first studies to examine the impact of MBSR on psychological well-being, diurnal salivary cortisol, and markers of inflammation in women veterans at risk for CVD. Furthermore, our RCT design was strengthened by the use of an ACC, which is often lacking in prior studies of MBSR.^41^ The attendance rate (54%) of six or more MBSR sessions was similar to the rate reported in a study examining MBSR in individuals with low back pain^42^ but lower than reported in a study of MBSR in newly diagnosed women with breast cancer.^16^ Sample characteristics, such as motivation to participate in MBSR, work and childcare commitments, and ability to travel to sessions may explain differences in attendance rates among studies.

Findings from our study demonstrate that women who participated in the MBSR program reported lowered perceived stress, loneliness, and symptoms of PTSD compared to those in the ACC. These findings are consistent with several previous studies reporting decreased perceived stress,^43,44^ loneliness,^17,45^ and PTSD symptoms^46,47^ following MBSR. However, unlike previous studies,^16,44^ we did not find significant improvements in depressive symptoms in the MBSR group compared with the ACC group. Although levels declined over time in both the MBSR and ACC groups, this decline was not significant.

It is noteworthy that the women who participated in MBSR reported significant increases in mindfulness as compared to those in the ACC group. These findings are consistent with other studies examining MBSR^16,48^ and demonstrate that MBSR was effective in improving mindfulness.

Women who participated in MBSR had a significant decline in salivary cortisol AUC compared to those in the ACC. Moreover, statistical analyses suggested that the cortisol trajectory for women in the MBSR group started to decline more rapidly 6 months post-intervention, whereas for women in the ACC group, cortisol began to rise. Dysregulated diurnal salivary cortisol is associated with an increased risk for mental and physical disorders, including metabolic disease.^49^ Previous studies have found mixed results related to salivary cortisol changes associated with MBSR with some studies reporting decreases in salivary cortisol following 4 weeks^19^ and 8 weeks of MBSR..^50^ Others reported no change in cortisol levels related to MBSR.^51^

We found no significant changes in IL-6 or IFN-γ production over time or differences between the MBSR and ACC groups. Only a few previous studies have assessed IL-6 and IFN-γ in relation to MBSR with some identifying a decrease in IL-6 and increase in IFN-γ production 1 month following an 8-week MBSR program^16^ and others finding no changes in IL-6.^52^ Differences in sample characteristics (sex, disease, etc.), measurement of cytokines (production versus circulating), and timing of sample procurement may account for these mixed results.

Several limitations are identified. Despite providing a detailed description of the study prior to enrollment, reminder phone calls prior to each class, and promptly calling participants who missed classes, the attrition rate was higher than we had anticipated. Although reasons for attrition varied, conflicts with work schedules and transportation difficulties were prominent. We recommend that for future studies, researchers consider examining MBSR using a variety of modes, including online and hybrid programs for women veterans. Furthermore, although we asked participants to keep diaries of their MBSR practice at home, we did not request that they submit the diaries to us. It may be beneficial to examine how MBSR home practice impacts outcomes in future studies. In addition, the PCL scores may represent general negative affect as it is unknown whether all participants experienced a stressor criterion for PTSD. Furthermore, we only measured combat exposure as a stressful life event. Future research should include other measures of traumatic stressful life events such as sexual trauma and physical assault. In addition, our findings are not generalizable to women who did not participate in the study due to lack of interest or other unknown reasons. Piloting MBSR in a pragmatic trial may provide additional insights into how MBSR can be used in routine clinical care as a method to help women veterans better manage their stress.

Despite limitations, our study has several strengths including a diverse sample with almost half (47%) of the sample identifying as non-White. Previously published studies examining MBSR have included mostly White samples.^53^ To our knowledge, this is the first RCT examining MBSR in an only-women veteran sample which previous studies have shown that women veterans prefer. Although more research is needed, offering MBSR programs specifically for women veterans in conjunction to their regular treatment may help women veterans better manage their stress and mental health. Making MBSR programs more accessible to women veterans by offering MBSR online and at varied times may help encourage women veterans to participate in the program.^23^ In addition, the VA Whole Health platform can be used to extend mindfulness to veterans through available videos, educational handouts, and VA apps.^54^ Other investigators have suggested offering MBSR to veterans in conjunction with other pleasurable activities, such as recreational sailing.^55^ Furthermore, providing MBSR training to clinicians within the VA may also allow MBSR to be offered more widely. Finally, educating clinicians, staff, and veterans on the benefits of MBSR may promote acceptance of the program as a method to reduce stress and improve health.

## References

[1] Han JK, Yano EM, Watson KE, Ebrahimi R (2019). Cardiovascular Care in Women Veterans. Circulation.

[2] Vimalananda VG, Miller DR, Christiansen CL, Wang W, Tremblay P, Fincke BG (2013). Cardiovascular disease risk factors among women veterans at VA medical facilities. J Gen Intern Med..

[3] Lehavot K, Goldberg SB, Chen JA, Katon JG, Glass JE, Fortney JC (2018). Do trauma type, stressful life events, and social support explain women veterans’ high prevalence of PTSD?. Social psychiatry and psychiatric epidemiology..

[4] Schnurr PP, Friedman MJ, Engel CC, Foa EB, Shea MT, Chow BK (2007). Cognitive behavioral therapy for posttraumatic stress disorder in women: a randomized controlled trial. Jama..

[5] McCauley HL, Blosnich JR, Dichter ME (2015). Adverse childhood experiences and adult health outcomes among veteran and non-veteran women. Journal of women's health (2002).

[6] Furman D, Campisi J, Verdin E, Carrera-Bastos P, Targ S, Franceschi C (2019). Chronic inflammation in the etiology of disease across the life span. Nat Med..

[7] Turner AI, Smyth N, Hall SJ, Torres SJ, Hussein M, Jayasinghe SU (2020). Psychological stress reactivity and future health and disease outcomes: A systematic review of prospective evidence. Psychoneuroendocrinology..

[8] Tyra AT, Soto SM, Young DA, Ginty AT (2020). Frequency and perceptions of life stress are associated with reduced cardiovascular stress-response adaptation. International journal of psychophysiology : official journal of the International Organization of Psychophysiology..

[9] **Levine GN, Lange RA, Bairey-Merz CN, Davidson RJ, Jamerson K, Mehta PK, et al.** Meditation and cardiovascular risk reduction: a scientific statement from the American Heart Association. J Am Heart Assoc. 2017;6(10). 10.1161/jaha.117.002218 10.1161/JAHA.117.002218 PMC5721815 28963100

[10] Momeni J, Omidi A, Raygan F, Akbari H (2016). The effects of mindfulness-based stress reduction on cardiac patients’ blood pressure, perceived stress, and anger: a single-blind randomized controlled trial. Journal of the American Society of Hypertension..

[11] Kabat-Zinn J (1990). Full Catastrophe Living.

[12] Keng SL, Smoski MJ, Robins CJ (2011). Effects of mindfulness on psychological health: a review of empirical studies. Clinical Psychology Review..

[13] Hilton L, Maher AR, Colaiaco B, Apaydin E, Sorbero ME, Booth M (2017). Meditation for posttraumatic stress: Systematic review and meta-analysis. Psychological trauma : theory, research, practice and policy..

[14] Khoury B, Sharma M, Rush SE, Fournier C (2015). Mindfulness-based stress reduction for healthy individuals: A meta-analysis. J Psychosom Res..

[15] Britton WB, Shahar B, Szepsenwol O, Jacobs WJ (2012). Mindfulness-based cognitive therapy improves emotional reactivity to social stress: results from a randomized controlled trial. Behavior therapy..

[16] Witek Janusek L, Tell D, Mathews HL (2019). Mindfulness based stress reduction provides psychological benefit and restores immune function of women newly diagnosed with breast cancer: A randomized trial with active control. Brain Behav Immun..

[17] Creswell JD, Irwin MR, Burklund LJ, Lieberman MD, Arevalo JM, Ma J (2012). Mindfulness-Based Stress Reduction training reduces loneliness and pro-inflammatory gene expression in older adults: a small randomized controlled trial. Brain Behav Immun..

[18] Kearney DJ, McDermott K, Malte C, Martinez M, Simpson TL (2012). Association of participation in a mindfulness program with measures of PTSD, depression and quality of life in a veteran sample. Journal of Clinical Psychology..

[19] Bergen-Cico D, Possemato K, Pigeon W (2014). Reductions in cortisol associated with primary care brief mindfulness program for veterans with PTSD. Med Care..

[20] Possemato K, Bergen-Cico D, Treatman S, Allen C, Wade M, Pigeon W (2016). A randomized clinical trial of primary care brief mindfulness training for veterans with PTSD. J Clin Psychol..

[21] Stephenson KR, Simpson TL, Martinez ME, Kearney DJ (2017). Changes in mindfulness and posttraumatic stress disorder symptoms among veterans enrolled in mindfulness-based stress reduction. J Clin Psychol..

[22] Marchand WR, Sandoval K, Lackner R, Parker SC, Herrmann T, Yabko B (2021). Mindfulness-based interventions for military veterans: A systematic review and analysis of the literature. Complementary therapies in clinical practice..

[23] Martinez ME, Kearney DJ, Simpson T, Felleman BI, Bernardi N, Sayre G (2015). Challenges to enrollment and participation in mindfulness-based stress reduction among veterans: a qualitative study. J Altern Complement Med..

[24] Witek-Janusek L, Albuquerque K, Chroniak KR, Chroniak C, Durazo-Arvizu R, Mathews HL (2008). Effect of mindfulness based stress reduction on immune function, quality of life and coping in women newly diagnosed with early stage breast cancer. Brain Behav Immun..

[25] Sephton SE, Salmon P, Weissbecker I, Ulmer C, Floyd A, Hoover K (2007). Mindfulness meditation alleviates depressive symptoms in women with fibromyalgia: results of a randomized clinical trial. Arthritis and rheumatism..

[26] Biegel GM, Brown KW, Shapiro SL, Schubert CM (2009). Mindfulness-based stress reduction for the treatment of adolescent psychiatric outpatients: A randomized clinical trial. Journal of Consulting & Clinical Psychology..

[27] Gross CR, Kreitzer MJ, Thomas W, Reilly-Spong M, Cramer-Bornemann M, Nyman JA (2010). Mindfulness-based stress reduction for solid organ transplant recipients: a randomized controlled trial. Alternative Therapies in Health & Medicine..

[28] Brown KW, Coogle CL, Wegelin J (2016). A pilot randomized controlled trial of mindfulness-based stress reduction for caregivers of family members with dementia. Aging Ment Health..

[29] Cohen S, Kamarck T, Mermelstein R (1983). A global measure of perceived stress. Journal of Health & Social Behavior..

[30] Radloff LS (1977). The CES-D Scale: A self-report depression scale for research in the general population. Applied Psychological Measurement..

[31] Russell DW (1996). UCLA Loneliness Scale (Version 3): reliability, validity, and factor structure. Journal of Personality Assessment..

[32] **Weathers FL, BT; Herman, D; Huska, J; Keane, T.** The PTSD Checklist-Civilian Version (PCL-C). National Center for PTSD, Boston MA. 1994.

[33] Witek-Janusek L, Gabram S, Mathews HL (2007). Psychologic stress, reduced NK cell activity, and cytokine dysregulation in women experiencing diagnostic breast biopsy. Psychoneuroendocrinology..

[34] Cutrona C, Russell D, Perlman WHJD (1987). Advances in personal relationships. Advances in personal relationships.

[35] Keane TM, Fairbank JA, Caddell JM, Zimering RT, Taylor KL, Mora CA (1989). Clinical evaluation of a measure to assess combat exposure. [References]. Psychological Assessment: A Journal of Consulting and Clinical Psychology..

[36] Baer RA, Smith GT, Hopkins J, Krietemeyer J, Toney L (2006). Using self-report assessment methods to explore facets of mindfulness. Assessment..

[37] Raudenbush SW, Bryk AS (2002). Hierarchical Linear Models: Applications and Data Analysis Methods.

[38] Hedeker D, Kaplan D (2004). An introduction to growth modeling. The SAGE handbook of quantitative methodology for the social sciences.

[39] Raudenbush SW, Liu XF (2001). Effects of study duration, frequency of observation, and sample size on power in studies of group differences in polynomial change. Psychological Methods..

[40] Singer J, Willett J (2003). Applied longitudinal data analysis: Modeling change and event occurence.

[41] Khusid MA, Vythilingam M (2016). The Emerging Role of Mindfulness Meditation as Effective Self-Management Strategy, Part 1: Clinical Implications for Depression, Post-Traumatic Stress Disorder, and Anxiety. Mil Med..

[42] Cherkin DC, Sherman KJ, Balderson BH, Cook AJ, Anderson ML, Hawkes RJ (2016). Effect of Mindfulness-Based Stress Reduction vs Cognitive Behavioral Therapy or Usual Care on Back Pain and Functional Limitations in Adults With Chronic Low Back Pain: A Randomized Clinical Trial. Jama..

[43] **Chen YH, Chiu FC, Lin YN, Chang YL.** The effectiveness of mindfulness-based-stress-reduction for military cadets on perceived stress. Psychol Rep. 2021:332941211010237. doi:10.1177/00332941211010237 10.1177/00332941211010237 33878969

[44] Gordon JL, Halleran M, Beshai S, Eisenlohr-Moul TA, Frederick J, Campbell TS (2021). Endocrine and psychosocial moderators of mindfulness-based stress reduction for the prevention of perimenopausal depressive symptoms: A randomized controlled trial. Psychoneuroendocrinology..

[45] O'Day EB, Butler RM, Morrison AS, Goldin PR, Gross JJ, Heimberg RG (2021). Reductions in social anxiety during treatment predict lower levels of loneliness during follow-up among individuals with social anxiety disorder. J Anxiety Disord..

[46] **Nugent SM, Freeman M, Ayers CK, Winchell KA, Press AM, O'Neil ME, et al.** A systematic review of therapeutic interventions and management strategies for gulf war illness. Mil Med. 2020. doi:10.1093/milmed/usaa260 10.1093/milmed/usaa260 33128563

[47] Davis LL, Whetsell C, Hamner MB, Carmody J, Rothbaum BO, Allen RS (2019). A multisite randomized controlled trial of mindfulness-based stress reduction in the treatment of posttraumatic stress disorder. Psychiatric research and clinical practice..

[48] Juul L, Pallesen KJ, Bjerggaard M, Nielsen C, Fjorback LO (2020). A pilot randomised trial comparing a mindfulness-based stress reduction course, a locally-developed stress reduction intervention and a waiting list control group in a real-life municipal health care setting. BMC Public Health..

[49] Golden SH, Sánchez BN, Wu M, Champaneri S, Diez Roux AV, Seeman T (2013). Relationship between the cortisol awakening response and other features of the diurnal cortisol rhythm: the Multi-Ethnic Study of Atherosclerosis. Psychoneuroendocrinology..

[50] Witek-Janusek L, Albuquerque K, Chroniak KR, Chroniak C, Durazo-Arvizu R, Mathews HL (2008). Effect of mindfulness based stress reduction on immune function, quality of life and coping in women newly diagnosed with early stage breast cancer. Brain, Behavior, & Immunity..

[51] Cash E, Salmon P, Weissbecker I, Rebholz WN, Bayley-Veloso R, Zimmaro LA (2015). Mindfulness meditation alleviates fibromyalgia symptoms in women: results of a randomized clinical trial. Ann Behav Med..

[52] Hecht FM, Moskowitz JT, Moran P, Epel ES, Bacchetti P, Acree M (2018). A randomized, controlled trial of mindfulness-based stress reduction in HIV infection. Brain Behav Immun..

[53] Szanton SL, Wenzel J, Connolly AB, Piferi RL (2011). Examining mindfulness-based stress reduction: perceptions from minority older adults residing in a low-income housing facility. BMC complementary and alternative medicine..

[54] Affairs USDoV. Whole Health: Reduce Stress through Mindfulness. 2020. https://www.va.gov/WHOLEHEALTH/features/Reduce_Stress_Through_Mindfulness.asp. Accessed 10/21/2021.

[55] Herrmann T, Marchand WR, Yabko B, Lackner R, Beckstrom J, Parker A (2020). Veterans' interests, perceptions, and use of mindfulness. SAGE open medicine..

